# Trade-offs between drug toxicity and benefit in the multi-antibiotic resistance system underlie optimal growth of E. coli

**DOI:** 10.1186/1752-0509-6-48

**Published:** 2012-05-25

**Authors:** Kevin B Wood, Philippe Cluzel

**Affiliations:** 1 Center for Systems Biology, Department of Molecular and Cellular Biology, and School of Engineering and Applied Sciences, Harvard University, Cambridge, MA, 02138, USA

## Abstract

**Background:**

Efflux is a widespread mechanism of reversible drug resistance in bacteria that can be triggered by environmental stressors, including many classes of drugs. While such chemicals when used alone are typically toxic to the cell, they can also induce the efflux of a broad range of agents and may therefore prove beneficial to cells in the presence of multiple stressors. The cellular response to a combination of such chemical stressors may be governed by a trade-off between the fitness costs due to drug toxicity and benefits mediated by inducible systems. Unfortunately, disentangling the cost-benefit interplay using measurements of bacterial growth in response to the competing effects of the drugs is not possible without the support of a theoretical framework.

**Results:**

Here, we use the well-studied multiple antibiotic resistance (MAR) system in *E. coli* to experimentally characterize the trade-off between drug toxicity (“cost”) and drug-induced resistance (“benefit”) mediated by efflux pumps. Specifically, we show that the combined effects of a MAR-inducing drug and an antibiotic are governed by a superposition of cost and benefit functions that govern these trade-offs. We find that this superposition holds for all drug concentrations, and it therefore allows us to describe the full dose–response diagram for a drug pair using simpler cost and benefit functions. Moreover, this framework predicts the existence of optimal growth at a non-trivial concentration of inducer. We demonstrate that optimal growth does not coincide with maximum induction of the *mar* promoter, but instead results from the interplay between drug toxicity and *mar* induction. Finally, we derived and experimentally validated a general phase diagram highlighting the role of these opposing effects in shaping the interaction between two drugs.

**Conclusions:**

Our analysis provides a quantitative description of the MAR system and highlights the trade-off between inducible resistance and the toxicity of the inducing agent in a multi-component environment. The results provide a predictive framework for the combined effects of drug toxicity and induction of the MAR system that are usually masked by bulk measurements of bacterial growth. The framework may also be useful for identifying optimal growth conditions in more general systems where combinations of environmental cues contribute to both transient resistance and toxicity.

## Background

The resistance of bacteria to antibiotics has prompted intense scientific research in the last several decades because it directly underlies the clinical treatment of infections [1]. While a large number of studies have focused on mutation-driven resistance, recent attention has also shifted to transient, or “inducible”, drug resistance taking place on much shorter timescales [2-9]. This transient resistance does not rely on mutations, but can be induced by a large class of chemicals commonly found in drugs (e.g. antibiotics and painkillers) and food preservatives. These chemicals are typically toxic to the cell when used alone, but they can also induce resistance to a broad range of agents. Consequently, they may prove beneficial to cells in the presence of multiple stressors. The net effect of a combination of chemical stressors can therefore be counterintuitive, because it is governed by the interplay between inducible resistance and drug toxicity. Such situations may arise, for example, in the human digestive tract, where bacteria face a cocktail of diverse chemical cues. The combined effects of multiple stressors, in general, have been studied for many decades in hopes of optimizing the clinical efficacy of combinatorial therapies [10-12]. More recently, the effects of drug interactions on the evolution of irreversible (mutation-driven) drug resistance have also been recognized [13-16]. Drugs that interact synergistically to produce a strong toxic effect can accelerate the acquisition of mutations conferring drug resistance [14]. On the other hand, antagonistic drug pairs produce a weaker toxic effect but can slow the acquisition of resistance [13,15]. These results demonstrate an inherent trade-off between the toxicity of the drug combination and its potential to facilitate drug resistance [17]. They also raise an interesting question: do trade-offs between drug toxicity and resistance also play a role in inducible drug resistance? Furthermore, when cells are exposed a combination of toxic agents that potentially induce transient resistance, how are these trade-offs related to synergy or antagonism between the given agents?

To address these questions, here we study inducible resistance mediated by the MAR (multiple antibiotic resistance) system. The MAR system, present in many bacterial species, consists of an operon that confers efflux-mediated [18-21] resistance to a broad range of antibiotics and can be activated by a host of chemical agents, including analgesics and food preservatives [2-5,7-9,22-25]. For example, in *E. coli*, high concentrations (5 mM) of salicylate, a well-known analgesic, lead to an increase in the minimum inhibitory concentration (MIC) of several antibiotics, and this effect has been partially attributed to the MAR system [4,26,27]. However, MIC alone masks the distinct effects of salicylate toxicity, antibiotic toxicity, and induction of the MAR system (the “benefit” of salicylate) on the growth, because it provides only an aggregate measure of these effects. Therefore, the underlying nature of the cost-benefit interplay remains hidden. Here we combine experiments with a simple phenomenological model to fully characterize a range of effects resulting from the trade-off between drug toxicity and the benefit mediated by efflux pumps.

## Results

### Experimental characterization of salicylate-induced antibiotic resistance

**Figure 1:**
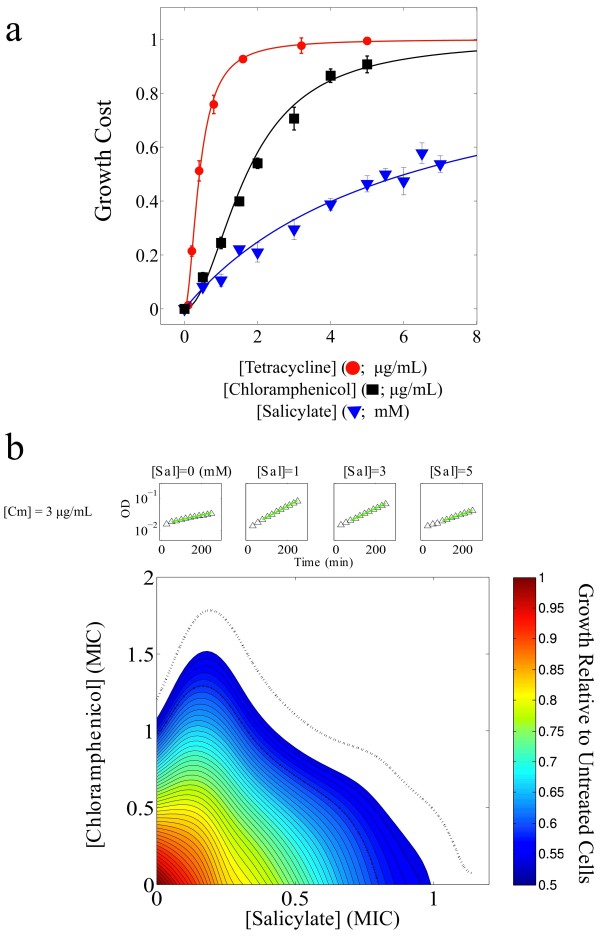
** Single and Multiple Drug Growth Costs.a**. Growth cost is defined as the reduction in growth rate of cells treated with a drug relative to the growth rate of untreated cells. Solid lines, best fits to Hill functions h(x) = x^n^/(K_i_^n^ + x^n^), with K_i_ equal to the concentration of drug i (i = S for salicylate, i = A for both antibiotics) at which growth is inhibited by 50%. K_A_ = 1.80 (1.66, 1.93) μg/mL, n = 1.97 (1.67, 2.26) for chloramphenicol; K_A_ = 0.41 (0.37, 0.45) μg/mL, n = 1.90 (1.61, 2.19) for tetracycline; and K_S_ = 6.06 (5.50, 6.62) mM, n = 1 for salicylate. 95% confidence intervals from nonlinear least squares fitting in parentheses. Error bars are standard deviation of four replicates. **b**. In the presence of high concentrations of chloramphenicol (Cm), growth is maximal for a nonzero concentration of salicylate (upper insets: OD time series). Contours of constant growth in concentration space indicate suppression. Dashed line, contours of 45% and 55% maximum growth.

To study the trade-off between drug toxicity and induced resistance, we first measured the effects of salicylate and two protein synthesis inhibitors, chloramphenicol and tetracycline, on cell growth. As expected, the effect of each drug alone is to slow cell growth as concentration increases. To quantitatively characterize this effect, we define growth cost as the reduction in growth rate of cells treated with one drug relative to the growth rate of untreated cells. In general, we find that cost functions are well-described by Hill functions with K_i_, the concentration of drug i at which cost is half maximal, and n, the Hill coefficient (Figure 1a). This mathematical form is consistent with standard dose–response models [10]. In the presence of a high concentration of chloramphenicol or tetracycline, however, we find that adding salicylate can increase growth (Figure 1b, Additional file 1: Figure S1). This effect was previously reported for a single concentration of salicylate [26]; here we provide the entire non-monotonic dependence of this suppressive effect on concentration, allowing us to tease apart contributions of salicylate’s cost from its benefit (below). We also performed detailed quantitative measurements of another suppressive interaction between an antibiotic, chloramphenicol, but with a different inducer, sodium benzoate (Additional file 1: Figure S1). 

### Phenomenological cost-benefit model for MAR-induced drug resistance

To quantitatively model the interplay between inducer cost and benefit in a two-drug environment that includes an inducer, we assume that the effects of the two drugs, in the absence of MAR induction, are independent in the sense that the relative growth rate of cells in the presence of both drugs (g_SA_) equals the product of individual relative growth rates of cells in the presence of each drug alone (g_SA_ = g_S_ g_A_). This assumption, known as Bliss independence [11], provides exact results for simple situations, such as a single enzymatic reaction disrupted by mutually non- exclusive inhibitors [10], in which case the effects of the drugs are defined in terms of reaction fluxes. However, the assumption of Bliss independence often fails for more complex systems, and it is therefore primarily used as a phenomenological null model for the physiological effects of non-interacting drugs on cell proliferation or growth [10,14,28-30].

**Figure 2:**
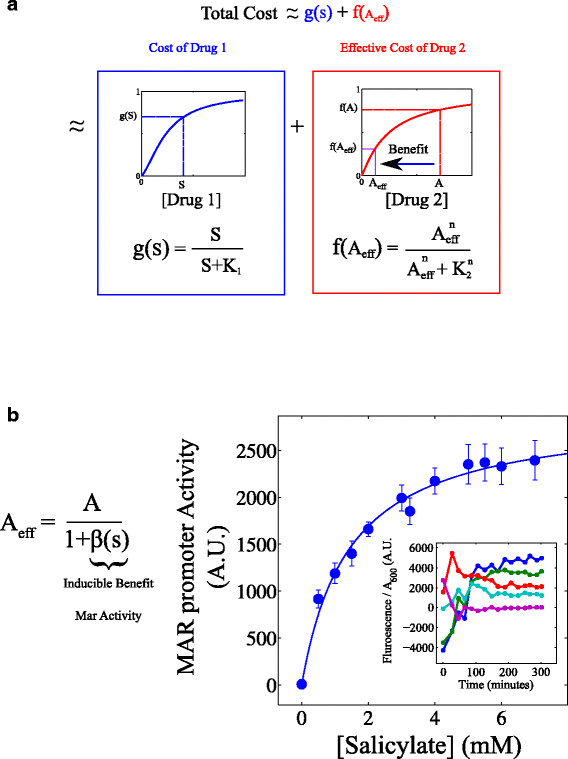
** Cost-Benefit Model for Interactions between MAR inducers and Antibiotics.a**. The combined growth cost of two drugs (S and A) is assumed to be Bliss independent, as long as the effective concentration of one drug is reduced by the presence of the second drug. Thus, the combined cost of both drugs is approximately equal to the sum of effective growth costs, f and g, for each individual drug. The reduction of each drug's growth cost depends on the concentration of the other drug, which in turn dictates the growth benefit provided. **b**. *mar* promoter activity, defined as the steady state time-averaged Fluorescence/OD times relative growth rate, increases as a function of salicylate concentration. Error bars are +/− one estimated standard deviation. Inset: mar fluorescence concentration (YFP fluorescence/optical density) time series for concentrations of salicylate ranging from 0 mM (purple) to 6 mM (blue). Data points are means over two replicates. Promoter activity is estimated by averaging fluorescence concentration in the steady state and multiplying by relative growth rate.

Here, we extend the concept of Bliss independence to include drug interactions mediated by the induction of the MAR system. Specifically, we assume that the presence of one drug (S) provides a fitness benefit by reducing the effective concentration of the second drug (A), thereby coupling the effects of the drugs. We incorporate this rescaling and re-write the model in terms of the combined growth cost, C_SA_ (by definition 1- g_SA_) to arrive at

(1) $$C_{\mathrm{SA}}=f\left(A_{\mathrm{eff}}\right)+g\left(s\right)-f\left(A_{\mathrm{eff}}\right)g\left(s\right)$$

where f(A) and g(S) are the *growth costs* of drugs alone (Figure 1a). Equation 1 expresses Bliss independence in terms of growth costs, and also generalizes it to include an S-dependent reduction of A to A_eff_. Therefore, the approximate additivity of drug costs implied by Bliss independence is modified to an approximate additivity of *effective* costs (Figure 2a). The model assumes that the presence of inducer reduces the effective concentration of drug A and adds an additional cost, but otherwise does not affect growth rate. This assumption considerably limits the spectrum of possible cellular responses to the drug pair, because drug S can only change the effective concentration of drug A, but will not change the shape of its cost function. The model can be readily extended to include two drugs which both act as inducers, in which case S is also changed to S_eff_. However, because the antibiotics used are poor inducers of the MAR system (Additional file 1: Figure S7), we neglect their effects on the concentration S and limit this study to the directional model implied by Equation [1].

A simple model, which assumes that the concentration of antibiotic A is reduced by the induction of efflux pumps (Additional file 1), suggests a form for A_eff_:

(2) $$A_{\mathrm{eff}}=\frac{A}{1+\beta \left(s\right)}$$

We call the function β(S) the *inducible benefit,* and it contains all quantitative information about the resistance mechanisms induced by drug S. To estimate the inducible benefit, β(S), for the MAR system, we measured the activity of the *mar* promoter as a function of S (Figure 2b; Additional file 1). MAR promoter activity could dictate the functional form for β(S) if, for example, we assume it is proportional to the number of efflux pumps produced in response to salicylate (Additional file 1). We scale this promoter activity by an adjustable parameter β_max_, so that $\beta (\text{S})\equiv \beta_{\text{max}}\;\text{S}/({\text{K}}_{\text{ind}}+\text{S})$, where K_ind_ = 0.8 is the concentration of inducer that yields half maximal promoter activity (Figure 2b). The adjustable parameter β_max_ links *mar* activity induced by drug S to a phenotypic response (resistance) to a second drug A. This parameter will be specific to drug A, and it provides a measure of the efficiency with which MAR induction eliminates A from the cell.

The cost-benefit theory assumes that the effects of the inducing drug and the antibiotic are independent, up to a reduction in the concentration of the antibiotic. The aim of our model is not to achieve a microscopic theoretical description of the system, but rather to provide a minimal phenomenological model that quantitatively captures the measured behavior. The success or failure of the model must therefore be determined experimentally. Specifically, our model does not attempt to elucidate the microscopic variables governing the multi-drug effects—which would require dozens, if not hundreds, of microscopic parameters—but rather posits that a simple relationship should exist between cell growth in the presence of one drug (which is a function of the cell’s internal state) and cell growth in the presence of two drugs (which is a function of an entirely different intracellular state). Specifically, the model requires equality between cell growth in the presence of A and cell growth in the presence of the combination S and A' (with A'> A), once we account for the costs of drug S. To directly verify this hypothesis, we can rearrange equation 1 to express this concept as

(3) $$\frac{C_{\mathrm{SA}}-g\left(S\right)}{1-g\left(S\right)}=f\left(A_{\mathrm{eff}}\right)$$

**Figure 3:**
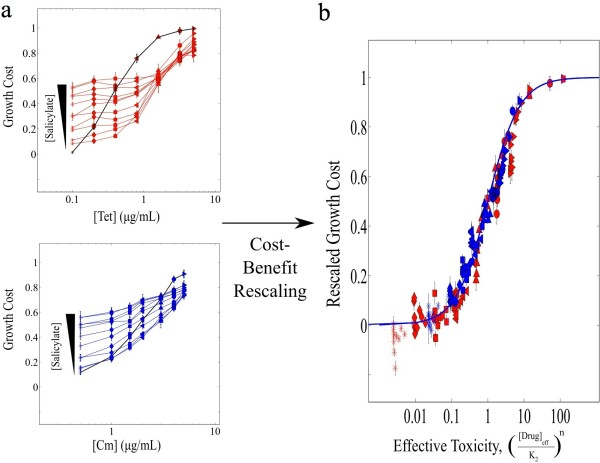
** Cost-Benefit Analysis Quantitatively Describes Multi-Drug Growth Cost Functions.a**. Multi-drug growth cost curves for salicylate and tetracycline (top) and salicylate and chloramphenicol (bottom) are increasing functions of antibiotic concentration, but also depend on the concentration of salicylate. Each growth cost curve represents growth in increasing concentrations of antibiotic at a single salicylate concentration, ranging from 0 to 7 mM. Black lines, ([salicylate] = 0 mM), which correspond to the single drug growth cost functions. Data points, means of four replicates. Error bars, +/− one sample standard deviation. **b**. The cost-benefit model requires rescaled versions of the multi-drug cost functions to have the same mathematical form as single drug cost functions (Equation 3). To directly test this assumption, we rescaled the multi-drug cost functions in panel **a** by removing the contribution from salicylate toxicity (cost) to each multi-drug growth cost function. Then, we rescaled the concentration of each antibiotic (chloramphenicol or tetracycline) using the single parameter β_max_, which accounts for the salicylate-induced reduction of intracellular antibiotic concentration (benefit). We trivially exploit the common Hill form of the single drug costs functions to show data from both drug pairs on a single plot, achieved by replacing drug concentration with effective toxicity, defined as ([A]/K_A_)^n^, where K_A_ and n characterize the single drug growth cost function for the antibiotics (see Figure 1). We use the salicylate induction curve of the *mar* promoter (Figure 2b) as the benefit function β(S), for both drug interactions, with only the overall scale of the benefit function (β_max_) specific to the two antibiotics. See also Additional file 1: Figure S2 for a direct comparison of growth rates from experiments and models.

The left hand side represents the "adjusted" multi-drug cost, once the toxic effects of S (cost) have been removed. The model implies that this adjusted cost of the drug combination, as a function of A_eff_, is functionally equivalent to the cost function f(x) for a single drug. Hence, if one removes from the multi-drug costs (C_SA_) the effects of g(S) according to the left-hand side of Equation 3 and then properly chooses the concentration reduction A→A_eff_ (determined by the single parameter β_max_), all two-drug data from a given drug combination should collapse to the single curve determined by f(x). Note that because a Hill function describes costs for many individual drugs as well as slices through many drug combination effect surfaces[10], collapsing the multi-drug cost curves from a given drug pair (Figure 3a) to a common Hill form can be simply achieved using independent parameters for each salicylate concentration. By contrast, equation 3 suggests that a non-trivial collapse of all curves for a given drug pair requires only a *single* parameter β_max_. Once this parameter is determined, the beneficial effects of different concentrations of salicylate are linked by the *mar* promoter activity curve. We verified this constraint (Figure 3b) by fitting the single free parameter, β_max_, using two-drug cost curves for salicylate and chloramphenicol (β_max_ = 1.15 +/− 0.15) and salicylate and tetracycline (β_max_ = 6.04 +/− 0.16). In all cases, the model provides an excellent fit to the data (R^2^ = 0.98; see Additional file 1: Figure S2 for a direct comparison of growth rates from experiment and model). Interestingly, this analysis demonstrates that a superposition of cost and benefit functions quantitatively describes the combined effects of salicylate and an antibiotic. Surprisingly, we find that the benefit function depends only on the inducer concentration and is dictated by the *mar* induction curve. 

### Transition from salicylate as toxic to salicylate as beneficial

**Figure 4:**
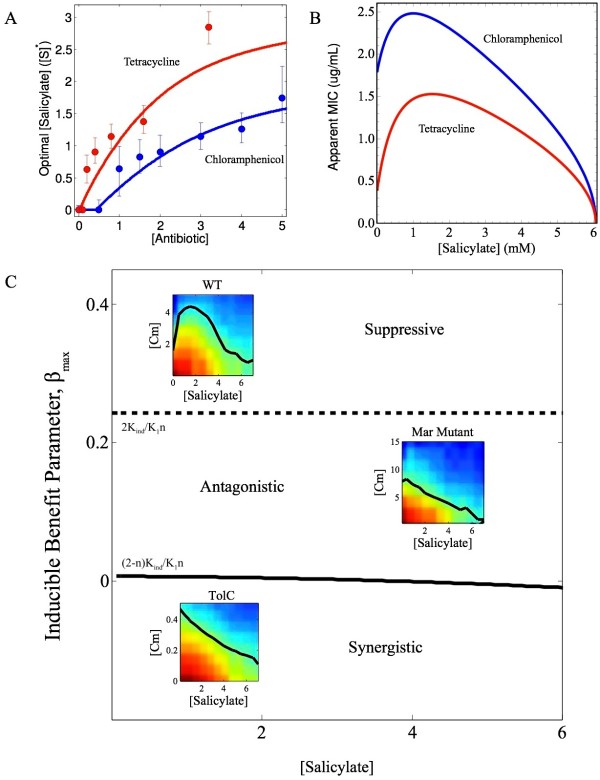
** Phase Diagram for Salicylate-Chloramphenicol Interactions.a**. The concentration of salicylate, S* (in mM), at which growth is maximal depends on the external concentration of antibiotic. A transition occurs between the low antibiotic regime, where the presence of salicylate is harmful, to the high antibiotic where the presence of salicylate increases the growth of cells. Red, tetracylcline; Blue, chloramphenicol. Concentrations for both drugs measured in μg/mL. Curves, numerical calculation from model; circles, estimates of S* from experiments at single concentrations of A. Maxima were estimated using cubic spline interpolation between data points at different concentrations of salcilyate. Error bars, concentrations corresponding to +/− 1% of maximum. **b**. Apparent MIC, defined as the minimum concentration of antibiotic (red, tetracycline; blue, chloramphenicol; both in units of μg/mL) at which growth is reduced to δ = 0.5. While the quantitative results depend on the precise definition of MIC, the qualitative features do not depend on the choice of δ. **c**. Cost-benefit theory predicts general properties of drug interactions as a function of the maximum inducible benefit β_max_ and the concentration of inducer, S. Solid line, phase boundary between synergistic and antagonistic drug interactions. Dashed line, phase boundary between antagonistic and suppressive interactions. In general, a higher value of the β_max_ increases the antagonism between drug pairs at a given concentration of drug S. Parameters characterizing individual drugs include K_S_ and K_ind_, which characterize, respectively, the cost of drug S and the corresponding induction of resistance systems (e.g. efflux pumps), and K_A_ and n, which characterize the cost of drug A. By contrast, β_max_ couples the individual effects of two drugs. Insets, heat maps of two-dimensional growth surfaces for 3 cell strains in the presence of Salicylate (Sal) and Chloramphenicol (Cm); top: β_max_ = 1.15 (WT cells; suppressive), β_max_ = 0.19 (mar mutant, antagonistic), and β_max_ = −0.15 (tolC mutant, synergistic). See also Additional file 1: Figure S3.

Our results uncover an interesting range of cellular behavior that emerges from the trade-offs of salicylate toxicity and the simultaneous induction of multi-drug resistance. First, for each concentration of antibiotic, we find that growth is maximal at a single salicylate concentration S*. In addition, we see an apparent transition between two regimes—one where the presence of the inducing drug is harmful, and another where it is beneficial—as A eclipses a threshold A_crit_ (Figure 4a). That is, S* becomes non-zero as A crosses A_crit_. In other words, the cell benefits from the presence of salicylate, but only when the antibiotic environment is sufficiently toxic to offset the inherent toxicity of the inducer. Interestingly, we find that *mar* promoter activity saturates at S ≈ 4 mM (Figure 2b), which is greater than S* for all concentrations of antibiotics used. Therefore, maximum suppression at S* results from a non-trivial interplay between MAR expression and inducer cost, not merely a maximum in MAR expression.

### Contributions of inducer cost and benefit to apparent MIC’s of antibiotics

Given our characterization of salicylate-induced multi-drug resistance, it is straightforward to calculate the apparent MIC of an antibiotic in the presence of any concentration of salicylate. Here, we define the apparent MIC to be the minimum concentration of drug A at which relative growth is reduced to a value of δ. For small concentrations of salicylate, the apparent MIC’s of both tetracycline and chloramphenicol increase dramatically (Figure 4b). However, at higher concentrations, the toxicity of salicylate begins to overwhelm its potential benefits, eventually leading to a decrease in apparent MIC. It is therefore clear that MIC reflects contributions of both the cost and benefits of the inducing drug. By contrast, the interdependence of inducible resistance and *mar* promoter activity can be described using only a single parameter, β_max_, which provides a quantitative measure of inducible benefit that is not masked by the effects of inducer cost.

### Phase diagram for MAR-mediated drug interactions

The interactions between salicylate and tetracycline, salicylate and chloramphenicol, and sodium benzoate and tetracycline are all suppressive, but this class of models describes a range of interactions ranging from synergistic to suppressive, based on the interplay of induction benefit and drug toxicity. Using Equations 1 and 2, we quantitatively determine a phase diagram (Figure 4, Additional file 1: Figure S3) that specifies how a drug interaction depends on the trade-offs between inducible benefit (described by the parameters K_ind_ and β_max_), the cost of inducer (K_S_), and the steepness of the antibiotic dose-response curve (n). Specifically, the cellular response to the first drug (S) alone includes both the growth cost of the drug (characterized by K_S_) and the induction of MAR system (characterized by K_ind_). The dynamics of the efflux pumps and their specificity for drug A determine the concentration reduction (A to A_eff_), which is governed by β_max_. The phase diagram demonstrates that properties of the drugs alone (n, K_S_, K_ind_) determine the level of drug coupling, contained in β_max_, required to achieve antagonism or suppression. Drugs that strongly induce growth benefit and have low associated cost (K_ind_ <<K_S_) are always suppressive. By contrast, high cost inducers (K_ind_ >> K_S_) can never be suppressive because the cost of induction is too high (Figure 4c).

The inducible benefit parameter (β_max_ = 1.15) characterizing the salicylate and chloramphenicol combination in wild type cells is far above the suppressive-antagonistic boundary (β_max_ > > 2K_ind_/(K_1_n) = 0.45), and the interaction is clearly suppressive for all concentrations of salicylate. To explore different regions of the phase diagram experimentally, we measured the effects of two different mutations on the suppressive drug interaction between salicylate and chloramphenicol. The first mutant, ΔtolC [31], lacks the protein TolC required for efflux pumping [32]. While we expect that the ΔtolC deletion will partially suppress the benefit induced by salicylate, we cannot predict how the costs of the drugs and, therefore, the precise nature of the drug interaction, will be altered. Experimentally, this mutant showed decreased resistance to chloramphenicol (Figure 4a, 4b), corresponding to a rescaling of the single drug cost (i.e. K_A_ smaller than in wild type), but the cost of salicylate remains unchanged (K_S_ approximately same as wild type). β_max_ was measured to be −0.15 +/− 0.01. According to the phase diagram (Figure 4), the interaction should therefore be weakly synergistic, and in fact the contours of constant growth appear slightly concave (lower inset). The suppressive drug interaction has been eliminated because the benefit associated with salicylate has been decreased while its cost remains unchanged, resulting in significantly less antagonistic interaction.

Since the suppressive interaction between salicylate and chloramphenicol is partially associated with the synthesis of AcrAB-TolC efflux pumps, we also hypothesized that cells with constitutive *mar* promoter activity would disrupt the cost-benefit interplay required for drug suppression. We selected such a mutant, here-called a *tet mutant*, by growing cells in 1 μg/mL tetracycline for 48 hours. We observed that the MAR system is a common target for mutations that confer resistance to tetracycline (see also [23]) (Additional file 1: Figure S6). In terms of our cost-benefit analysis, these cells synthesize resistance systems (benefit) without the associated cost of an inducing drug. To verify the molecular basis of resistance in the tet mutant, we sequenced the full genome and found only a single point mutation in the α5 region of the MAR repressor marR, which is linked to dimer formation and subsequent binding to the *mar* promoter [33]. In the salicylate-chloramphenicol combination, the tet mutant showed increased resistance to chloramphenicol (K_A_ is larger than in wild-type) and a near-additive drug interaction (β_max_ = 0.19 +/− 0.03) between salicylate and chloramphenicol (Figure 4). Because the cost of salicylate is unchanged, the elimination of suppression suggests that the mutation has blocked the inducible benefit of salicylate (see also Additional file 1: Figure S5 for similar results with a different drug pair). Biologically, the inactivation of the MarR repressor no longer requires the presence of salicylate, and therefore the cost of the drug exceeds its associated inducible benefit. Interestingly, we also found that the *mar* activity in the mutant is higher in the absence of salicylate than the maximum *mar* activity induced by even high concentrations of salicylate in the wild type strain. However, adding salicylate further increases *mar* activity in the mutant (Additional file 1: Figure S7A). Moreover, the functional dependence of the induction on salicylate is similar to that of the wild-type (Additional file 1: Figure S7A, inset). This result is not intuitive because the beneficial effects of salicylate on the growth of the mutant in the presence of chloramphenicol are quite small (β_max_ is approximately 17% of that of the wild type, and the antagonism between the drugs is markedly decreased).

## Discussion

Efflux is a widespread cellular defense mechanism with clinical consequences for antimicrobial treatments. Cost-benefit principles provide a convenient tool for analyzing these ubiquitous systems. We have shown that such a framework provides a quantitative description of drug interactions associated with the classic MAR system, which mediates an inducible resistance to many different toxic agents. The model allows us to quantify the relative importance of the induction mediating reversible resistance and the toxicity of the inducing agent. In particular, we observe a transition between two regimes, one where salicylate is harmful and one where it is beneficial, as the concentration of antibiotic crosses a threshold A_crit_. By disentangling the contributions of inducer cost and benefit, the analysis also provides a quantitative measure (β_max_) of the reversible (MAR-mediated) resistance to each antibiotic used. This measure complements previous MIC measurements [26], which provide information about the net effect of both inducer costs and benefits but that mask the relative contributions of each. We demonstrate that it is precisely this balance between cost and benefit that determines whether the antimicrobial properties of multiple agents are synergistic, antagonistic, or suppressive. Therefore, in addition to providing a new quantitative model of the MAR system, we also lay the groundwork for a link between the short-term trade-offs of inducible resistance, which determine the type of interaction between pairs of toxic agents, and the acquisition of irreversible resistance mediated by these drug interactions.

This framework may be applicable to other systems where environmental signals can be toxic but also induce transient resistance. For example, cancer cells can activate pro-survival stress responses upon treatment with chemotherapies or radiation[34], and subpopulations of tumor cells can access new transcriptional/differentiation states to become reversibly drug resistant[35]. Our analysis implements efflux-mediated resistance using a concentration reduction A **→** A_eff_ for the effective concentration of drug A, and this form could be used to describe other common mechanisms of resistance, including enzymatic decay and target modification. As a consequence, the cost-benefit framework developed here may prove useful for modeling and understanding a wide range of systems governed by the inherent trade-offs of inducible resistance.

## Conclusions

Our analysis provides a quantitative description of the MAR system and demonstrates that optimal cell growth in the presence of an inducer and an antibiotic does not coincide with maximum induction of the *mar* promoter, but instead results from the interplay between drug toxicity and *mar* induction. In addition, we show that this interplay determines whether the drugs are synergistic, antagonistic, or suppressive. The framework may also be extensible to more general systems where combinations of environmental cues contribute to both transient resistance and toxicity.

## Methods

### Strains

We used the *E. coli* Frag-1B strain, which is wild-type for the AcrAB multiple drug efflux pump system, and also the wild-type *E.coli* BW25113 strain. The ΔtolC mutant is strain JW5503-1 from the Keio collection.

### Drugs

Drug solutions were made from solid stocks (sodium salicylate, Sigma-Aldrich; chloramphenicol, MP Biomedicals; tetracycline hydrochloride, Acros Organics; doxycycline hyclate, Sigma- Alderich; kanamycin, Fisher; ciprofloxacin, Sigma-Alderich). All antibiotic stock solutions were stored in the dark at −20°C. All drugs were thawed (when necessary) and diluted in sterilized Lennox LB broth (Fisher) for experimental use.

### Growth conditions and drug treatments

We inoculated LB media with a single colony and grew the cells overnight (12 h at 30°C with shaking at 200 rpm). Following overnight growth, stationary phase cells were diluted (600–1000 fold) in LB media containing 0.2% glucose and grown for an additional 3 h (30°C with shaking at 200 rpm). Following the initial dilution period, we transferred cells to 96 well microplates (round bottom, polystyrene, Corning) and set up a two-dimensional matrix of drug concentrations in each 96-well microplate (165 μl media per well). For the remainder of the experiment (4–8 h), cells were grown at 30°C with shaking at 1200 rpm on a Heidolph Titramax vibrating microplate shaker (Brinkmann). Plates were wrapped in aluminum foil to minimize evaporation. A600 (absorbance at 600 nm, proportional to optical density OD) and YFP fluorescence were measured at 15–25 min intervals for 4–8 h using a Wallac Victor-2 1420 Multilabel Counter (PerkinElmer).

### Growth rate estimation and MIC determination

Growth rates were determined by fitting multiple regions of growth curves (A_600_ vs. time, Additional file 1: Figure S4) in early exponential phase to an exponential function (MATLAB 7.6.0 curve fitting toolbox, The Mathworks) using a variable length (45–150 minutes) sliding window. Growth rate corresponded to the window position associated with best fit to an exponential function, determined by a minimum root mean squared error (RMSE). For each experimental condition, the final growth rate was the sample mean of 4 replicates (See also Additional file 1). Growth rate contours were estimated from experimental data using the Matlab *csaps* function to interpolate between data points (typically 96) spaced roughly evenly throughout the two dimensional space of drug concentrations. MIC was taken to be the drug concentration(s) at which growth was inhibited by 50%.

### Drug resistant mutants

We diluted liquid cultures of wild-type cells (in stationary phase) 1000-fold into 96 individual 150 μL cultures on a single microplate, with each well supplemented with 1.0 μg/mL tetracycline. After approximately 48 h of growth at 30°C with shaking, optical density and fluorescence were measured from each well. Samples from randomly selected cultures exhibiting high OD and fluorescence (Additional file 1: Figure S6) were frozen in 15% glycerol at −80°C. Frozen cultures were used to streak LB plates, and all experiments (including sequencing) were performed on cultures grown from a single colony isolated from these plates.

## Competing interests

The authors declare that they have no competing interests.

## Authors’ contributions

KW and PC designed research, analyzed data, and wrote the paper. KW performed all experiments. Both authors read and approved the final manuscript.

## Supplementary Material

Additional file 1 **Supplemental Figures, Supplemental Methods, and Supplemental Notes [**[10,23,36-42]**]. ** Click here for file
