# Supplementary material for: Trade-offs between drug toxicity and benefit in the multi-antibiotic resistance system underlie optimal growth of E. coli
Source: BMC Syst Biol. 2012 May 25;6:48. doi: 10.1186/1752-0509-6-48 (PMC3462682; doi:10.1186/1752-0509-6-48)
Supplement: Additional file 1 — Supplemental Figures, Supplemental Methods, and Supplemental Notes [[10,23,36-42]]. [file 1752-0509-6-48-S1.pdf]

Supplemental Figures

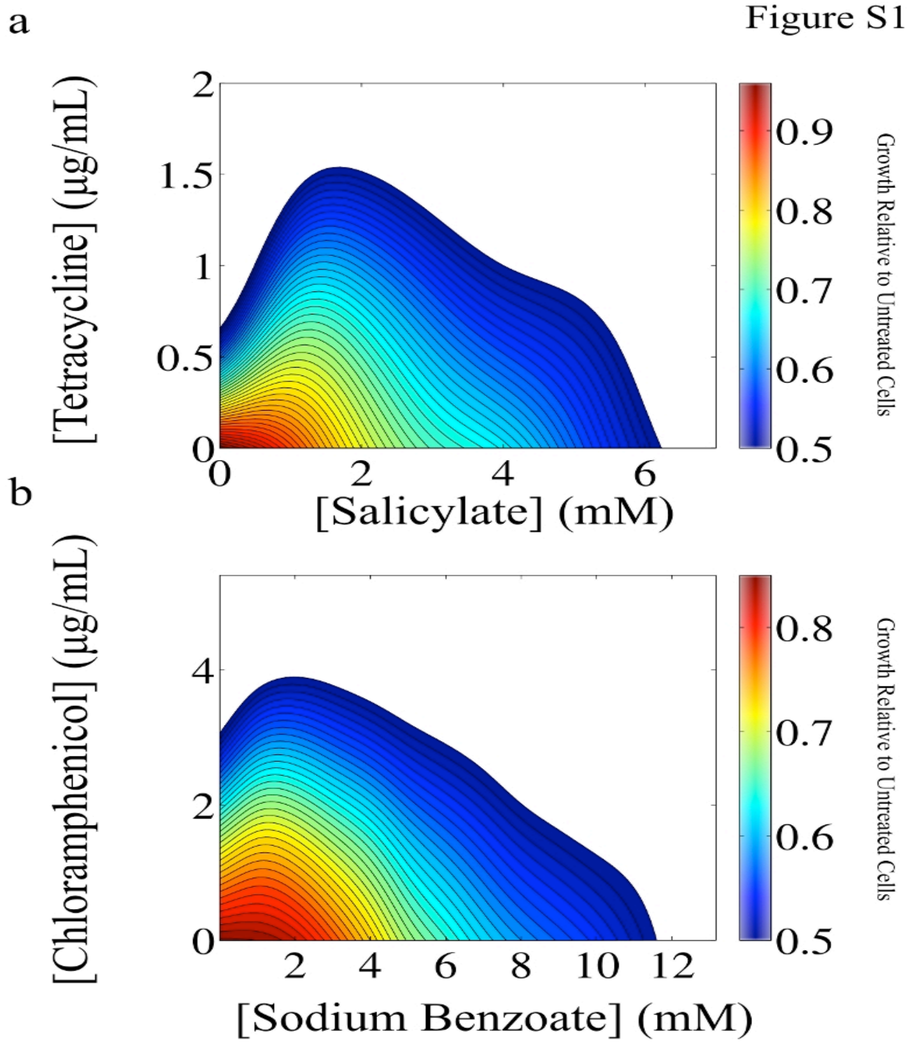

Figure S1: Suppressive Drug Interactions Occur Between Salicylate and Tetracycline and Between Sodium Benzoate and Chloramphenicol

a. Salicylate interacts suppressively with tetracycline. Growth rate was estimated by fitting time series of absorbance A_{600} covering early exponential phase growth to an exponential function using a variable length sliding window. Growth rate contours are determined by cubic spline interpolation of 96 approximately equally separated data points in tetracycline-salicylate space. Each data point is a mean of four replicates.

b. Sodium benzoate interacts suppressively with chloramphenicol. To estimate growth rate, stationary phase cell cultures were diluted 1000x at time t_1 and grown in LB media supplemented with various drugs concentrations until time t_2 = 9-12 hours. Based on the dilution factor (1000) and the final optical density, an average growth rate was estimated as $k \approx \log\left(OD_{t_2} / OD_{t_1}\right) / \left(t_2 - t_1\right)$, where OD_t is the optical density at time t. Growth rate contours are determined by cubic spline interpolation of 48 approximately equally separated data points in chloramphenicol-sodium benzoate space. Each data point is a mean of two replicates.

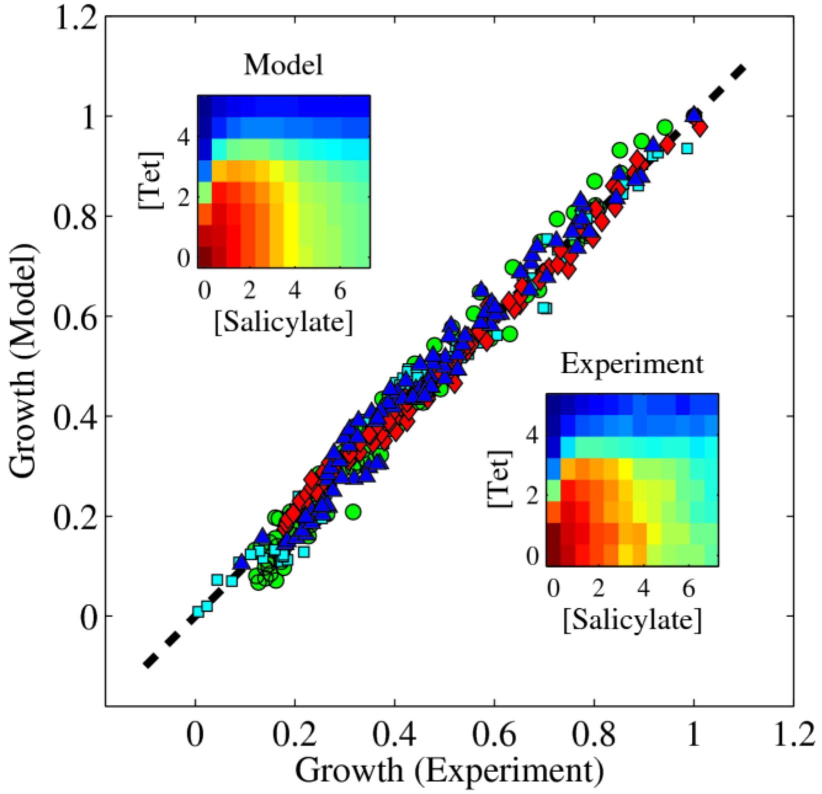

Figure S2: Cost-Benefit Theory Provides a Quantitatively Accurate Model for Interactions Between Salicylate and Antibiotics in Both Wild Type (WT) and Mutant Cells

Blue triangles, Sal-Cm (WT Cells); Red diamonds, Sal-Tet (WT Cells), Cyan squares, Sal-Cm (TolC mutant); Green circles, Sal-Cm (Tet mutant). Insets, heat map of growth (dark blue, 0; red, 1) for both the model (upper left) and experiment (lower right) for wild type cells exposed to the Sal-Tet combination. $R^2 > 0.95$ in all cases.

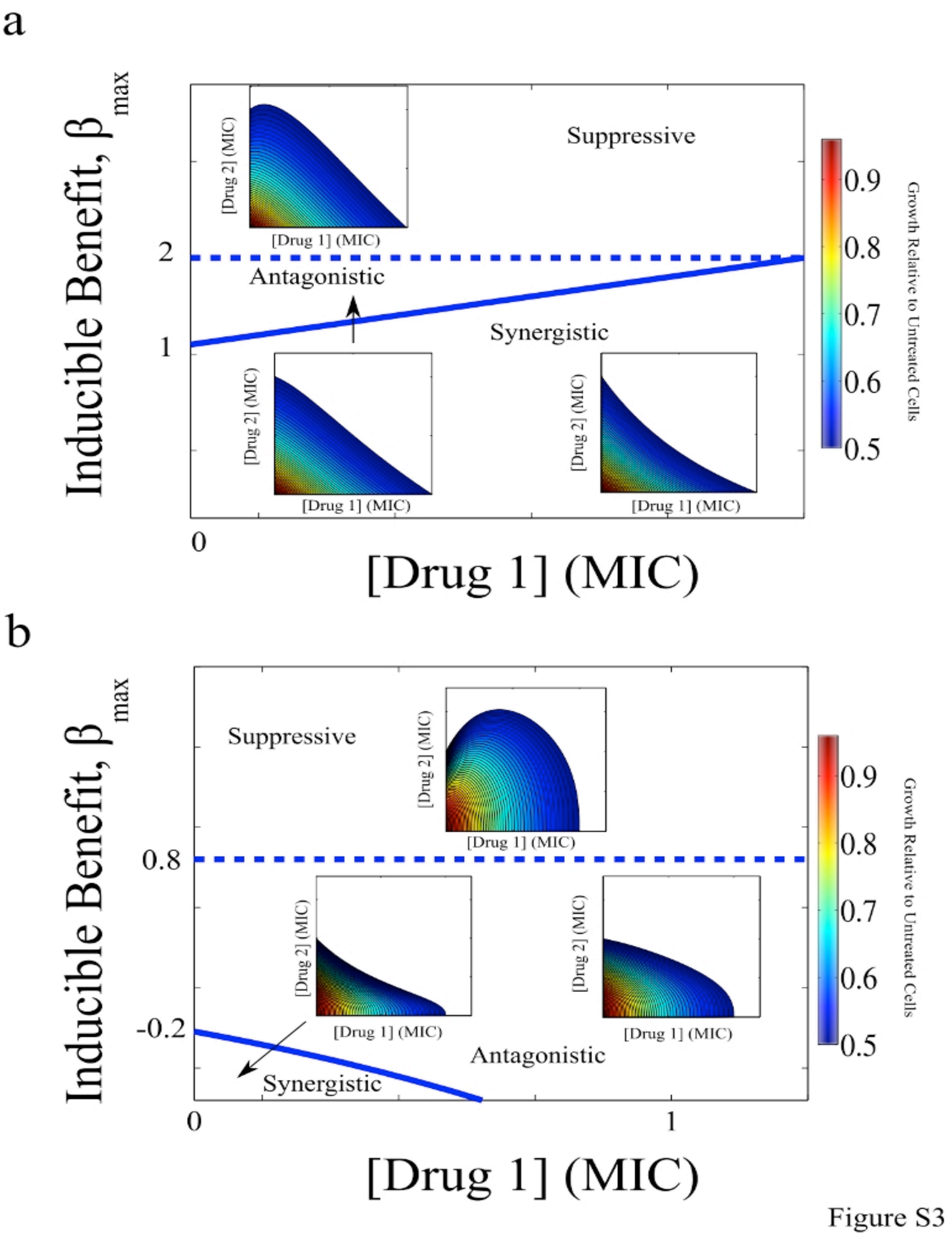

Figure S3: Example Theoretical Phase Diagrams for Different Values of n, the Hill Coefficient for Drug Cost

a. Phase diagram with $K_1=K_2=K_{ind}=n=1$. Boundary between antagonistic and suppressive

interactions is given by $\beta_{max} = 2\ K_{ind}/K_1\ n = 2$. Line separating antagonistic and suppressive interactions intersects the vertical axis at $\beta_{max} = (2-n)\ K_{ind}/K_1\ n = 1$. Example contour plots are shown for $\beta_{max} = 0.5$ (lower right), 1.5 (lower left), 3 (upper). Note that drug interactions appear synergistic even in the presence of nonzero inducible benefit.

b. A. Phase diagram with $K_1=K_2=K_{ind}=1$ and n=2.5. Boundary between antagonistic and suppressive interactions is given by $\beta_{max} = 2\ K_{ind}/K_1\ n = 0.8$. Line separating antagonistic and suppressive interactions intersects the vertical axis at $\beta_{max} = (2-n)\ K_{ind}/K_1\ n = -0.2$. Example contour plots are shown for $\beta_{max} = -0.9$ (lower left), 0.5 (lower right), 3 (upper).

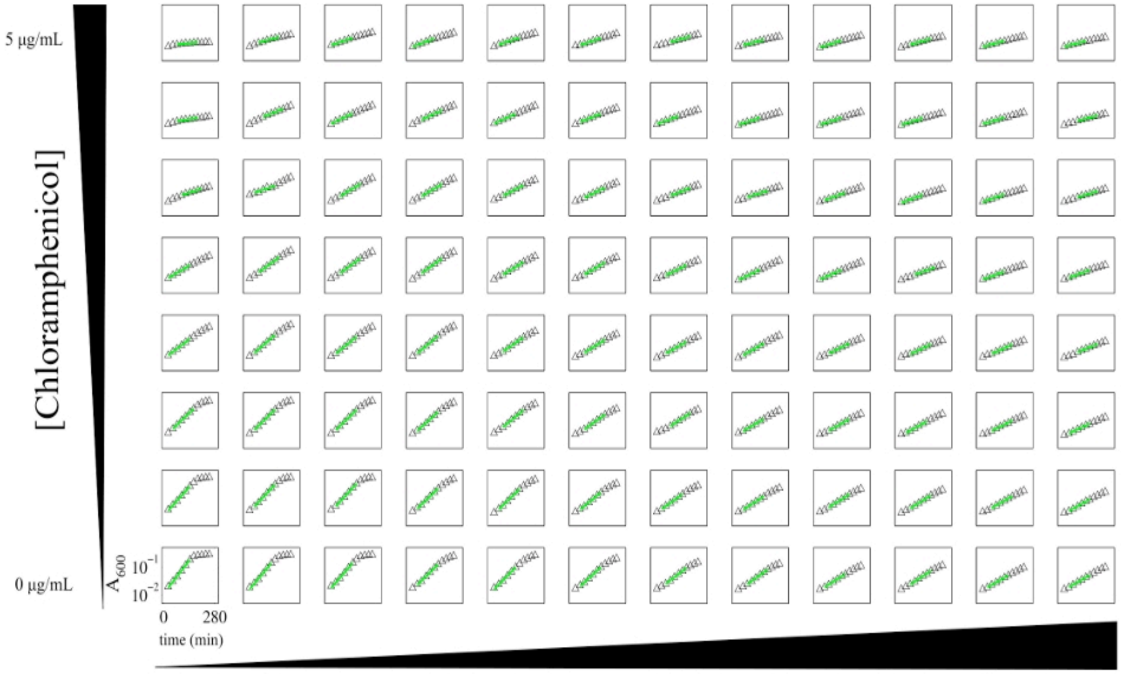

Figure S4

Figure S4: Growth curves for Salicylate and Chloramphenicol.

Time series of absorbance A_{600} for cells grown in combinations of chloramphenicol and salicylate. [Salicylate] (left to right) = 0, 0.5, 1.5, 2, 3, 4, 5, 5.5, 6, 6.5, 7 mM; [chloramphenicol] (bottom to top) = 0, 0.5, 1, 1.5, 2, 3, 4, 5 μg/mL. Solid lines, best fits to exponential functions.

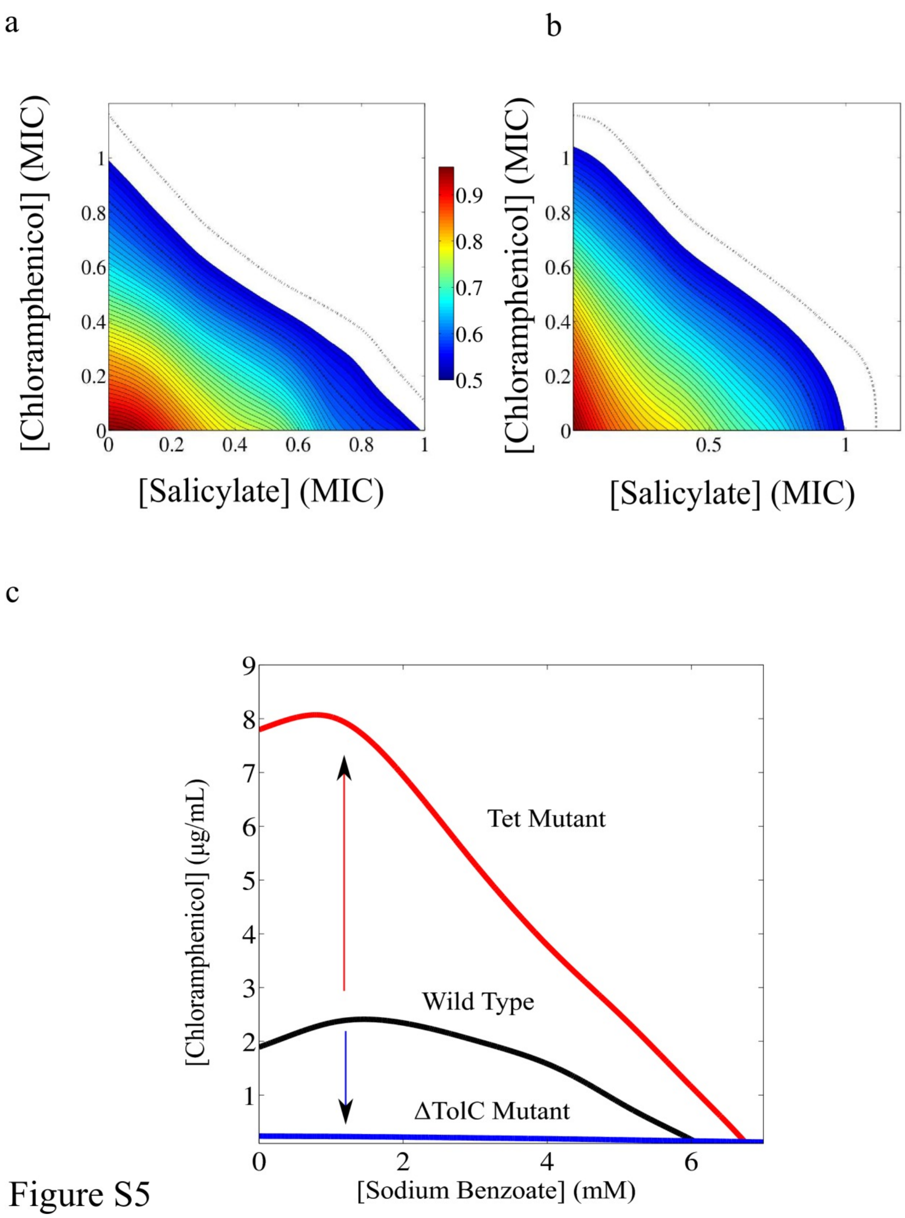

Figure S5

Figure S5: Mutations Decrease Suppression Between Salicylate and Chloramphenicol and Between Sodium Benzoate and Chloramphenicol.

a. Contour plot of growth rate of ΔTolC mutant exposed to salicylate and chloramphenicol (see also phase diagram in Figure 4).

b. Contour plot of growth rate of tetracycline mutant exposed to salicylate and chloramphenicol (see also phase diagram in Figure 4).

c. M.I.C. contours for sodium benzoate and chloramphenicol indicate suppressive interactions on wild type cells (black line), weak antagonism on tetracycline-selected Mar mutants, and no suppression on ΔTolC mutants. M.I.C. contours were estimated by the growth contour approximately half way between the minimum and maximum estimated growth rate (see caption for Figure S1b).

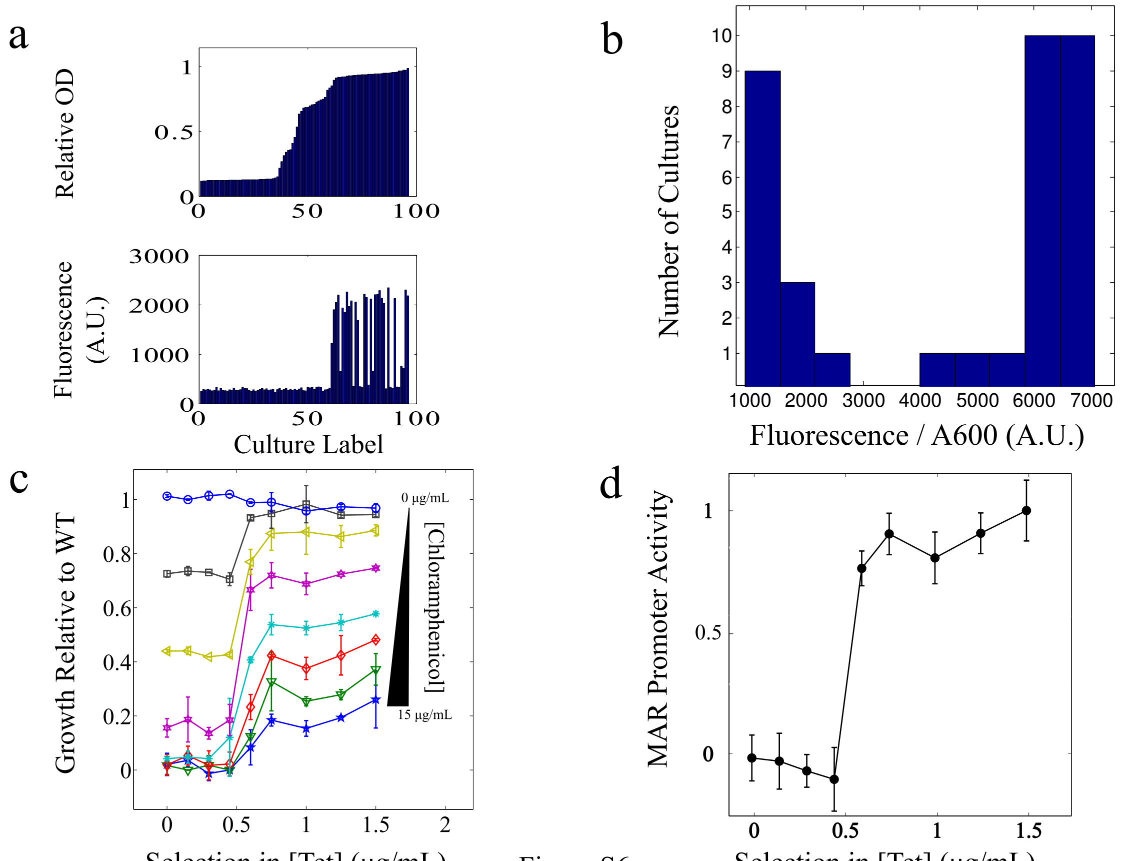

Figure S6

Figure S6: Cells Adapt to Long-Term Drug Exposure by Developing Resistance in the Absence of Inducer

a. Ninety six independent cultures were grown in the presence of 1.0 μg/mL of tetracycline. Optical density (top) and fluorescence (bottom) of each culture are measured after 48 hours and sorted by optical density. Approximately 35% of the cultures exhibit sufficient resistance to reach stationary phase in the allotted time (top), and many of these cultures show largely increased fluorescence due to the *mar* promoter (bottom).

b. Of the 35 cultures that developed significant resistance and grew to stationary phase, 22 had significantly increased *mar* promoter activity as indicated by increased fluorescence concentration (greater than 3000 A.U.), suggesting that resistance often arises from a nearly cost-free mutation which up-regulates MAR activity.

c. Cells grown for approximately 48 hours in [tetracycline] > 0.5 ug/mL develop resistance to chloramphenicol and tetracycline. Curves, relative growth of adapted cells in the presence of increasing concentrations of chloramphenicol. In the absence of drugs, the adapted cells suffer little or no fitness growth cost relative to wild-type cells (top blue curve). Data points, means of 2 replicates. Error bars extend to max and min of replicates.

d. *mar* promoter activity as a function of [tetracycline] used for selection. Cells selected by [tetracycline] > 0.5 μg/mL show significantly increased *mar* promoter activity, even in the absence of inducing drugs. Error bars, +/- one standard deviation of (Fluorescence/OD) fluctuations in steady state. Data normalized so that maximum activity is 1

A

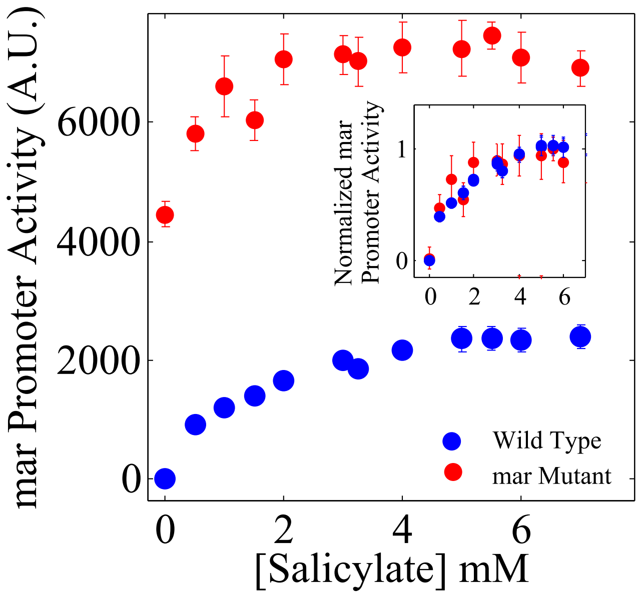

B

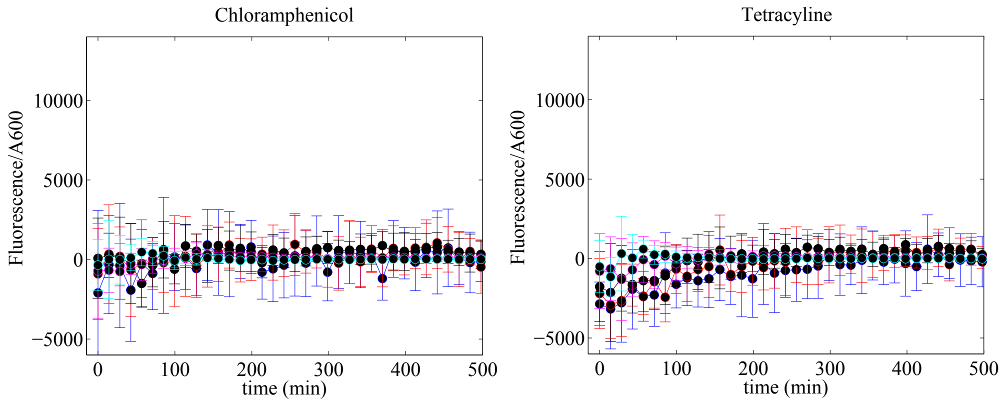

Figure S7

Figure S7: Salicylate significantly increases *mar* promoter activity, but tetracycline and chloramphenicol do not. A. Salicylate increases *mar* activity in wild type (blue) and tet mutant

(red) cells, and the dose dependence is similar in both strains (inset). B. Fluorescence concentration (Fluorescence/A_{600}) time traces are shown for wild type cells grown in the presence of Cm (top) and Tet (bottom). The different colors correspond to the concentrations given below. Top panel: blue (16 μg/mL), red (8 μg/mL), black (4 μg/mL), pink (1.3 μg/mL), light blue (0 μg/mL); Bottom panel: blue (3 μg/mL), red (1.5 μg/mL), black (0.75 μg/mL), pink (0.25 μg/mL), light blue (0 μg/mL). It is clear that Tet and Cm are much weaker inducers of the mar system than salicylate, which strongly induces the mar system after approximately 100 minutes, even at concentrations (< 2 mM) that only slightly decrease growth (compare to inset, Figure 2b). Error bars represent sample standard deviations from 6 independent trials.

Supplemental Methods

Tetraclycine Resistant Mutants

To select for tetracycline-resistance MAR mutants, we grew 96 individual 150 μl cultures of wild type cells in a high concentration (1 μg/mL) of tetracycline. After 48 h, we randomly chose a culture that grew to stationary phase and also exhibited increased *mar* promoter activity (as measured by YFP expression) relative to wild type cells. We subsequently isolated a single mutant, here called a Tet-mutant, by selecting one colony from the culture.

While this randomly selected mutant with constitutive *mar* promoter activity eliminated suppression between salicylate and chloramphenicol (Figure 4b), it was not clear how commonly similar mutations affecting the MAR system arise following antibiotic exposure. However, it is well-known that mutations related to the MAR system can be selected by tetracycline[1,2]and such mutations have also been isolated from clinical samples[3,4,5]. To verify the prevalence of *mar* promoter activity in our selection experiments, we measured the growth and *mar* promoter activity of the remaining 95 cultures grown in 1 μg/mL tetracycline. After 48 h, 40 of the cultures had reached stationary phase (Fig. S6a). These cultures presumably contained the resistant mutants which would dominate a large culture, and over half (22/35) showed substantially

increased fluorescence, corresponding to high *mar* promoter activity (Fig. S6b). To verify the significance of mutations affecting the MAR system in larger cultures and characterize the cross resistance of such mutants to chloramphenicol, we grew 3 mL cultures of wild-type cells for 48 h in various concentrations of tetracycline up to approximately 3 times the MIC. Adapted cells grown in tetracycline concentrations greater than 0.5 μg/mL grew at the same rate as wild type cells and developed cross resistance to chloramphenicol (Fig. S6c). In addition, these resistant cells showed high levels of *mar* promoter activity (Fig. S6d). Thus, the MAR system was a common target of resistance-conferring mutations in cells exposed to tetracycline. This result suggests that, similar to the Tet mutant, cells grown in high levels of tetracycline for several days adapt to exploit the resistance conferred by the MAR system without the associated cost and toxicity of an inducing drug.

Measuring mar Promoter Activity

To monitor *mar* promoter activity, we used the YFP reporter plasmid pZS*2 MAR-venus[6-8], which contains the *mar* promoter as well as Kan^r and a SC101* ori that maintains the copy number at 3-4 copies/cell.

Mar promoter activity was determined by first correcting raw YFP fluorescence by subtracting a background fluorescence curve (fluorescence vs. absorbance) obtained from untreated cells. Temporal profiles of *mar* background-corrected fluorescence concentration (fluorescence/absorbance) were generated from means of two replicates (Fig. 2). *Mar* promoter activity was taken to be the background-corrected fluorescence concentration (fluorescence/absorbance), averaged over steady state, times the growth rate k. Fluorescence concentration alone is a sufficient measure of relative promoter activity in strains with similar growth rates.

Supplemental Notes:

Drug degradation model for inducible benefit

To determine a functional form for A_{eff}, we assume that the internal antibiotic concentration a is governed by

$$\dot{a} = k_1\left(A - a\right) - \left(k_2 + \Delta k_2\right)a \qquad \text{(S1)}$$

where A is the external concentration of antibiotic, k_1 is the rate constant governing passive influx of drug into the cell, k_2 is the rate constant governing drug degradation and/or efflux in the absence of inducer, and Δk_2 captures the change in drug degradation activity imparted by the presence of inducer. By definition, $\Delta k_2 = 0$ in the absence of inducer. Equation S1 assumes that dilution from cell growth is slow on the timescale of efflux pumping and can therefore be neglected. In the steady state, the internal concentration a is a function of Δk_2,

$$a_{ss}\left(\Delta k_2\right) = \frac{A}{\left(1 + k_2/k_1 + \Delta k_2/k_1\right)} \qquad \text{(S2)}$$

Equation S2 suggests that we define an effective antibiotic concentration A_{eff} as

$$\frac{A_{eff}}{A} \equiv \frac{a_{ss}\left(\Delta k_2\right)}{a_{ss}\left(0\right)} = \frac{1}{1 + \frac{\Delta k_2}{\left(k_1 + k_2\right)}} \qquad \text{(S3)}$$

With this definition, equation S2 simplifies to

$$a_{ss}\left(\Delta k_2\right) = \frac{A_{eff}}{\left(1 + k_2/k_1\right)} \qquad \text{(S4)}$$

Since $\Delta k_2/(k_1+k_2)$ is assumed to contain the entire dependence on inducer concentration S, we can generalize S3 by writing

$$A_{eff} = \frac{A}{1 + \beta\left(S\right)} \qquad \text{(S5)}$$

where $\beta(S)$ is defined as the inducible benefit. While the functional form of $\beta(S)$ can, in general, be arbitrarily complex, physical arguments suggest that inducible benefit will be a saturating function of S. In the case of the MAR system, we experimentally verify that the model can quantitatively describe several multi-drug combinations if $\beta(S)$ is taken to be proportional to the normalized activity of the *mar* promoter, with β_{max} a scaling constant equal to the asymptotic value of β as $S \rightarrow \infty$ (Figure 3, Figure S2). Equivalently, we are assuming that the increase in efflux rate Δk_2 is proportional to the relative *mar* promoter activity. Such proportionality would be expected, for example, if the *mar* promoter activity was proportional to the number of efflux pumps synthesized in response to inducer. More generally, the form S5 captures the notion that increasing inducible benefit $\beta(s)$ decreases the effective concentration A_{eff}.

The model S1 is not critical to our overall hypothesis, but we nevertheless note some of its limitations. First, we do not account for dilution of intracellular antibiotic by cell growth. Second, we assume that the internal antibiotic concentration can be used to approximate the internal "free" (unbound) antibiotic concentration. We make the preceding two assumptions to reduce the number of parameters and simplify the interpretation of our experiments, but we cannot rule out more complex behavior in other experimental regimes. Relaxing these two assumptions gives rise to a much more complex situation. For example, in cases where cell permeability (passive influx) is very low, there is the possibility of bistable growth. While we never observed any experimental evidence of such bistability, this possibility has been considered in more detailed theoretical models [9]. Our rescaling model is a specific instance of this more general model that includes bistability.

Derivation of general phase diagram

To derive a phase diagram, we begin with the definition of Lowe additivity of two drugs, which says[5]

$$\frac{S_\delta}{S_{0,\delta}} + \frac{A_\delta}{A_{0,\delta}} = 1 \tag{S6}$$

where S_δ and A_δ are the concentrations of drug 1 and 2, respectively, in a mixture that results in a fractional growth inhibition δ. Similarly, $S_{0,\delta}$ and $A_{0,\delta}$ are the concentrations of drugs 1 and 2 alone that result in a growth inhibition δ. For simplicity, in what follows we take δ = 1/2; that is, we define the drug interaction based on the contour line in drug concentration space defined by 50% growth inhibition. In this case, $S_{0,\delta}$ and $A_{0,\delta}$ reduce to K_1 and K_2, respectively, which are the binding constants characterizing the single drug cost functions. Deviations from this additivity result in synergy (left hand side of Equation S6 < 1) or antagonism (left hand side of Equation S6 > 1).

The contour separating drug synergy from antagonism--that is, the contour of additivity--can be found in the (S, β_{max}) space by setting κ = 1/2 in Equation 3, using Equations 1,2, 4 and 5 to solve for $A_{1/2}$, and then plugging into Equation S6. The contour separating synergy from antagonism is then given by

$$\beta_{max} = \left(\frac{K_{ind}+S}{S}\right)\left(\left(\frac{K_1+S}{K_1}\right)\left(\frac{K_1-S}{K_1+S}\right)^{\frac{n-1}{n}} - 1\right) \tag{S7}$$

While the shape of the phase boundary will, in general, depend on the specific parameters, it is straightforward to show that

$$\lim_{S\to 0}\beta_{max} = \frac{K_{ind}}{K_1}\left(\frac{2}{n}-1\right) \tag{S8}$$

meaning that the phase boundary intersects the vertical axis at a value of β_{max} proportional to K_{ind}/K_1. In general, for a given value of S, increasing β_{max} beyond a threshold given by S7 will lead to a transition from synergistic to antagonistic interactions. Interestingly, there is a range of β_{max} values for which the nature of the drug interaction depends on S (Fig. S3).

Drug suppression is an extreme form of antagonism where the effect of two drugs is less than that of one drug alone. In our simple model with drug 1 chosen to be an inducing drug, suppression will arise when a maximum exists at S=S* in the the growth contour in drug concentration space and, additionally, A(S*) > A(0). Using Equations 1-5, it is straightforward to show that the slope $\frac{\partial A}{\partial S}$ characterizing the contour of constant growth (50%) in drug concentration space is a monotonically decreasing function of S. Furthermore, it is clear that the contour A(S) approaches zero at S = K_1, the MIC. In order for a maximum in the contour A(S) to exist, it is therefore necessary and sufficient that

$$\lim_{S \to 0} \frac{\partial A}{\partial S} = K_2 \left(\frac{\beta_{max}}{K_{ind}} - \frac{2}{K_1 n} \right) > 0 \qquad \text{(S9)}$$

Any maximum will have A(S*) > A(0). We therefore have the following additional condition for drug suppression:

$$\beta_{max} > \frac{2\mathrm{K}_{ind}}{K_1 n} \qquad \text{(S10)}$$

Generally, the nature of a drug interaction is determined by a balance between the cost of the physiological response, which determines the phase boundaries, and the benefit conferred by this response, which is governed by β_{max}. Specifically, the phase boundary separating antagonism from suppression depends on the ratio K_{ind}/K_1 , where the constant K_1 characterizes the inducer cost and K_{ind} the induction of physiological components which potentially provide benefit. The ratio K_{ind}/K_1 therefore measures the cost of inducing beneficial elements in response to drug 1. In addition, the phase boundary for suppression decreases with increasing n, the Hill coefficient governing the steepness of the antibiotic cost function. For large n>>1, the antibiotic cost

function approaches a step function. Therefore, even a slight shift in drug concentration can result in a significant benefit, as the cost drops abruptly from 1 to 0 as concentration is decreased across the threshold value K_2. As a result, the onset of suppression requires only a nonzero β_{max}. However, in practice, n is typically on the order of 1, so the phase boundary is not significantly dependent on n. Two examples of phase diagrams for different values of n are shown in Figure S3. While the phase boundary separating synergy from antagonism depends on n, in all cases increasing β_{max} at a given concentration S leads to increasingly antagonistic and eventually suppressive behavior.

Supplmental References

1. Cohen, S., McMurry, L., Hooper, D., Wolfson, J. & Levy, S. Cross-resistance to fluoroquinolones in multiple-antibiotic-resistant (Mar) Escherichia-coli selected by tetracycline or chloramphenicol: decreased drug accumulation associated with membrane changes in addition to OmpF reduction. *Antimicrobial Agents and Chemotherapy* **33**, 1318-1325 (1989).
2. George, A.M. & Levy, S.B. Amplifiable resistance to tetracycline, chloramphenicol, and other antibiotics in Escherichia coli: involvement of a non-plasmid-determined efflux of tetracycline. *J. Bacteriol* **155**, 531-540 (1983).
3. Alekshun, M.N. & Levy, S.B. Regulation of chromosomally mediated multiple antibiotic resistance: the mar regulon. *Antimicrob. Agents Chemother* **41**, 2067-2075 (1997).
4. Maneewannakul, K. & Levy, S. Identification of mar mutants among quinolone-resistant clinical isolates of Escherichia coli. *Antimicrob. Agents Chemother* **40**, 1695-1698 (1996).
5. Greco, W.R., Bravo, G. & Parsons, J.C. The search for synergy: a critical review from a response surface perspective. *Pharmacol. Rev* **47**, 331-385 (1995).
6. Guet, C., Bruneaux, L., Oikonomou, P. & Cluzel, P. *In preparation* (2011).

7. Le, T.T., Emonet, T., Harlepp, S., Guet, C.C. & Cluzel, P. Dynamical determinants of drug-inducible gene expression in a single bacterium. *Biophys. J* **90**, 3315-3321 (2006).
8. Lutz, R. & Bujard, H. Independent and tight regulation of transcriptional units in Escherichia coli via the LacR/O, the TetR/O and AraC/I1-I2 regulatory elements. *Nucleic Acids Res* **25**, 1203-1210 (1997).
9. Elf, J., Nilsson, K., Tenson, T. & Ehrenberg, M. Bistable bacterial growth rate in response to antibiotics with low membrane permeability. *Phys. Rev. Lett* **97**, 258104 (2006).
